# Left and right atrial appendage functional features as predictors for voltage-defined left atrial remodelling in patients with long-standing persistent atrial fibrillation

**DOI:** 10.1007/s00380-020-01752-4

**Published:** 2021-01-02

**Authors:** Radoslaw Marek Kiedrowicz, Maciej Wielusinski, Andrzej Wojtarowicz, Jaroslaw Kazmierczak

**Affiliations:** Cardiology Department, Pomeranian Medical University, Powstancow Wlkp. 72, 70-111 Szczecin, Poland

**Keywords:** Atrial fibrillation, Atrial appendage, Long-standing persistent atrial fibrillation, Voltage mapping, Electrical remodelling, Low-voltage areas

## Abstract

It was hypothesised that left atrial (LA) fibrosis identified by the presence of low-voltage areas (LVA) may influence the mechanical and electrical function of the left (LAA) and right (RAA) atrial appendage among the long-standing persistent atrial fibrillation (LSPAF) population. 140 consecutive patients underwent voltage mapping of LA with a multielectrode catheter following pulmonary vein isolation and restoration of sinus rhythm with cardioversion. Echocardiography determined LAA peak outflow and inflow velocities and intracardiac catheter-based mean LAA and RAA AF cycle length (AFCL) were obtained during AF before ablation. The impact of flow velocities and AFCL on the prevalence and location of LVA was further evaluated. LVA were detected in 54% of the patients. 14% of the patients presented severe global LVA burden > 20% of the total LA surface area. 29% of the patients presented a disseminated pattern of remodelling as 3 out of 5 LA segments were affected. LAA AFCL, RAA AFCL, LAA flow velocities did not predict the absolute presence of LVA. However LAA AFCL > 155 ms predicted disseminated LVA pattern and LAA AFCL > 165 ms severe LVA incidence. LAA AFCL > 155 ms was predictive for existence of LVA within antero-septal LA segments whilst LAA emptying velocity ≤ 0.2 m/s within lateral wall. Moreover RAA AFCL > 165 ms was strongly related to the presence of LAA AFCL > 15 ms and > 165 ms. LAA and RAA functional assessment was predictive of the presence of advanced stages of voltage-defined LA fibrosis and its regional distribution among LSPAF population

## Introduction

Left atrial (LA) fibrosis, that can be identified by the presence of low-voltage areas (LVA), is thought to play an important role in the development and maintenance of atrial fibrillation (AF) [1]. Some studies have shown evidence of LVA with several markers, however, with conflicting results, especially among the long-standing persistent AF (LSPAF) population [2]. Therefore, parameters predicting LVA burden still remain unclear. It was observed that left atrial appendage (LAA) remains intact despite the development of voltage-defined fibrosis in the body of LA [3, 4], recently confirmed in LSPAF patients [2]. Hence, we hypothesised that the presence of LVA may influence the mechanical and electrical function of the left and right (RAA) atrial appendage. Therefore, we decided to evaluate the impact of LAA flow velocities along with intracardiac LAA and RAA AF cycle length (AFCL) on the prevalence and location of LVA in LSPAF population detected with high-density and high-resolution bipolar voltage mapping.

## Methods

### Study population

One hundred and sixty-three consecutive LSPAF patients who underwent RF point-by-point catheter ablation were prospectively enrolled at our centre. The patients with a history of AF ablation procedure or any cardiac surgery, severe valvular disease or mechanical valve, previous myocarditis or pericarditis, LAA thrombus detection (*n *= 22) and having been given amiodarone within the last 6 months were excluded. All antiarrhythmic drugs were discontinued for at least five half-lives before ablation. The study protocol was approved by a local institutional review board and all patients provided written informed consent.

### Echocardiography examination

Transthoracic and transoesophageal echocardiography (TOE) were performed before ablation using a Vivid E9 ultrasound system (GE Vingmed Ultrasound AS). Standard left atrial and ventricular (LV) parameters were measured, determined according to the recent recommendations [5].

LAA peak outflow (emptying) and inflow (filling) velocities were measured during AF on TOE with pulsed wave Doppler placed in close proximity to the LAA orifice. All patients had well-controlled heart rate (< 90 bpm) to ensure adequate quality images. All values for each parameter were averaged over three to five successive cardiac cycles. Low LAA flow velocities were defined as ≤ 0.2 m/s [6]. LAA long-axis views were used to manually calculate its maximum depth measured from the ostium to the most distal visualised lobe.

### AFCL measurement

**Fig. 1:**
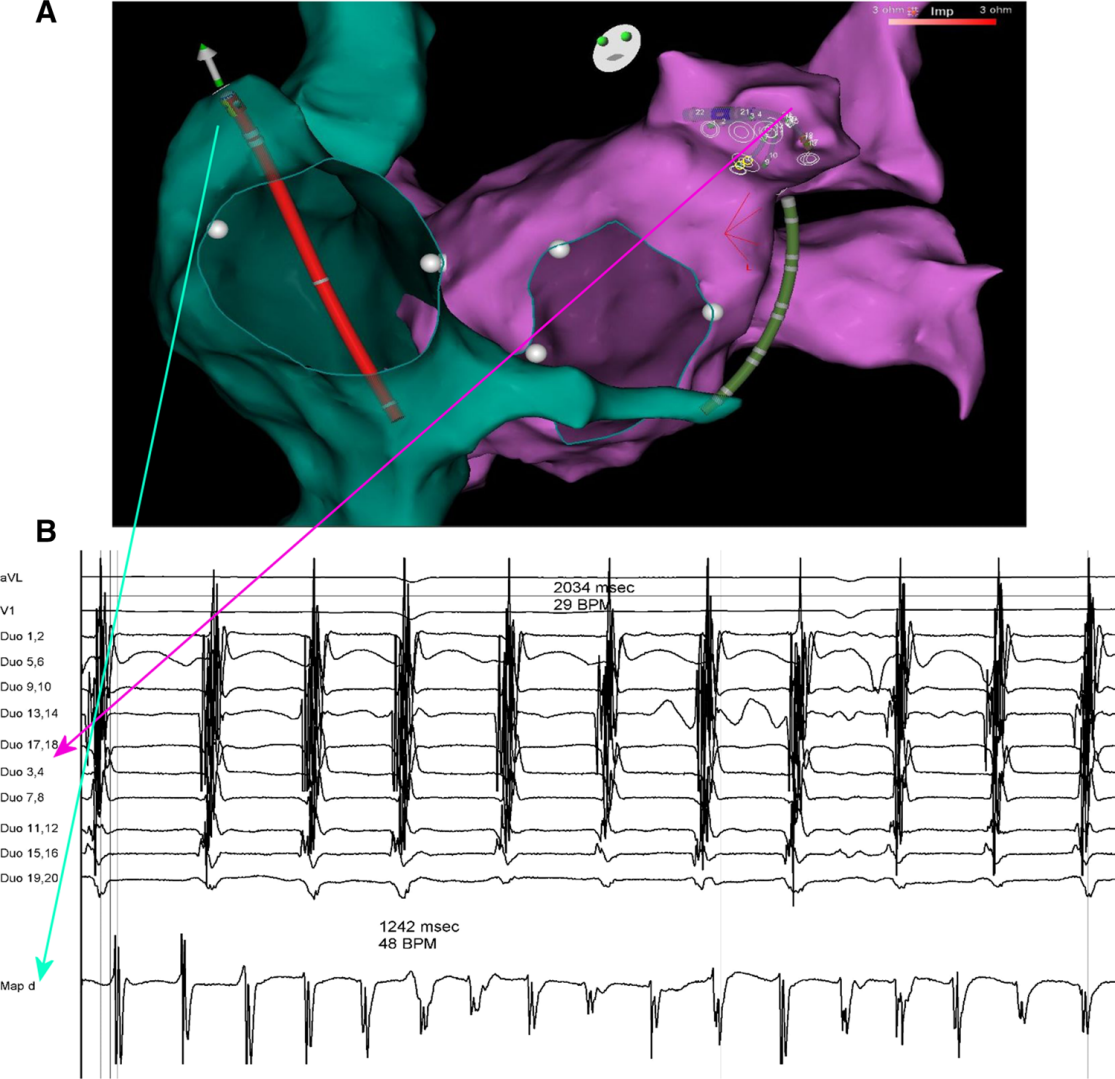
Atrial fibrillation cycle length measurement within the right and left atrial appendage. The figure shows a Thermocool SmartTouch catheter positioned within the right atrial appendage (RAA) and a Pentaray catheter positioned within the left atrial appendage (LAA), displayed on the reconstructed atrial shells acquired with the CARTO®3 electroanatomical platform (panel A). Intracardiac electrograms recorded with CardioLab electrophysiology system were used for a mean AF cycle length CL (AFCL) calculation in each appendage by averaging the CL of 10 consecutive beats at a sweep speed of 100 mm/s using electronic callipers (panel B). Note that in this case the mean RAA AFCL (124,2 ms) is shorter than a mean LAA AFCL (203,4 ms)

Following the introduction of a Thermocool SmartTouch catheter (Biosence-Webster, BW) to the RA, it was advanced into the RAA. A Pentaray duodecapolar catheter (BW) was positioned in the LAA immediately after transseptal access. Both catheters were manipulated until receiving local electrograms of an amplitude > 0.5 mV and an organised AF pattern defined as the lack of multicomponent atrial electrograms with fractionation, baseline perturbation with continuous atrial activity and short AF cycle lengths < 120 ms. The proper position of the catheters within appendages was confirmed on the reconstructed atrial shells with the CARTO®3 electroanatomical platform (BW). Intracardiac electrograms were recorded using CardioLab electrophysiology system (GE Healthcare). A mean AFCL was calculated in each appendage by averaging the CL of 10 consecutive beats with signal filtering set at 30–300 Hz in review mode at a sweep speed of 100 mm/s using electronic callipers (Fig. 1). The mean total AFCL (average of the mean LAA and RAA AFCL) and AFCL gradient (LAA AFCL divided by the RAA AFCL) were prospectively calculated.

### Detection of LVA

Only patients who were able to maintain sinus rhythm following pulmonary vein isolation (PVI) and cardioversion (*n* = 140) underwent high-density high-resolution LA bipolar voltage mapping [2963(2213–3145) points per map] using the CARTO®3 system. The mapping protocol is described in detail elsewhere [2]. Briefly, this was performed during coronary sinus (CS) pacing with a Pentaray catheter, acquired with a CONFIDENSE™ module (BW). The voltage map was created during CS pacing to reduce the occurrence of spontaneous atrial ectopy and to facilitate the identification of incorrectly annotated points. To ensure detailed mapping, the distance filling threshold was set at 5 mm, the tissue proximity filter was always enabled and only mapping sites that were within a distance of 5 mm from the acquired shell contributed to the voltage map. Further discrete mapping using a SmartTouch catheter, which covered less than 10% of the total LA surface area (TSA), at sites presenting inadequate Pentaray-tissue contact was performed if necessary. Electrograms were only accepted if a contact force was ≥ 6 g. EGM amplitude ≥ 0.5 mV was defined as normal and < 0.5 mV as diseased tissue. All points presenting low voltage were visually inspected and those incorrectly annotated were deleted from the map.

**Fig. 2:**
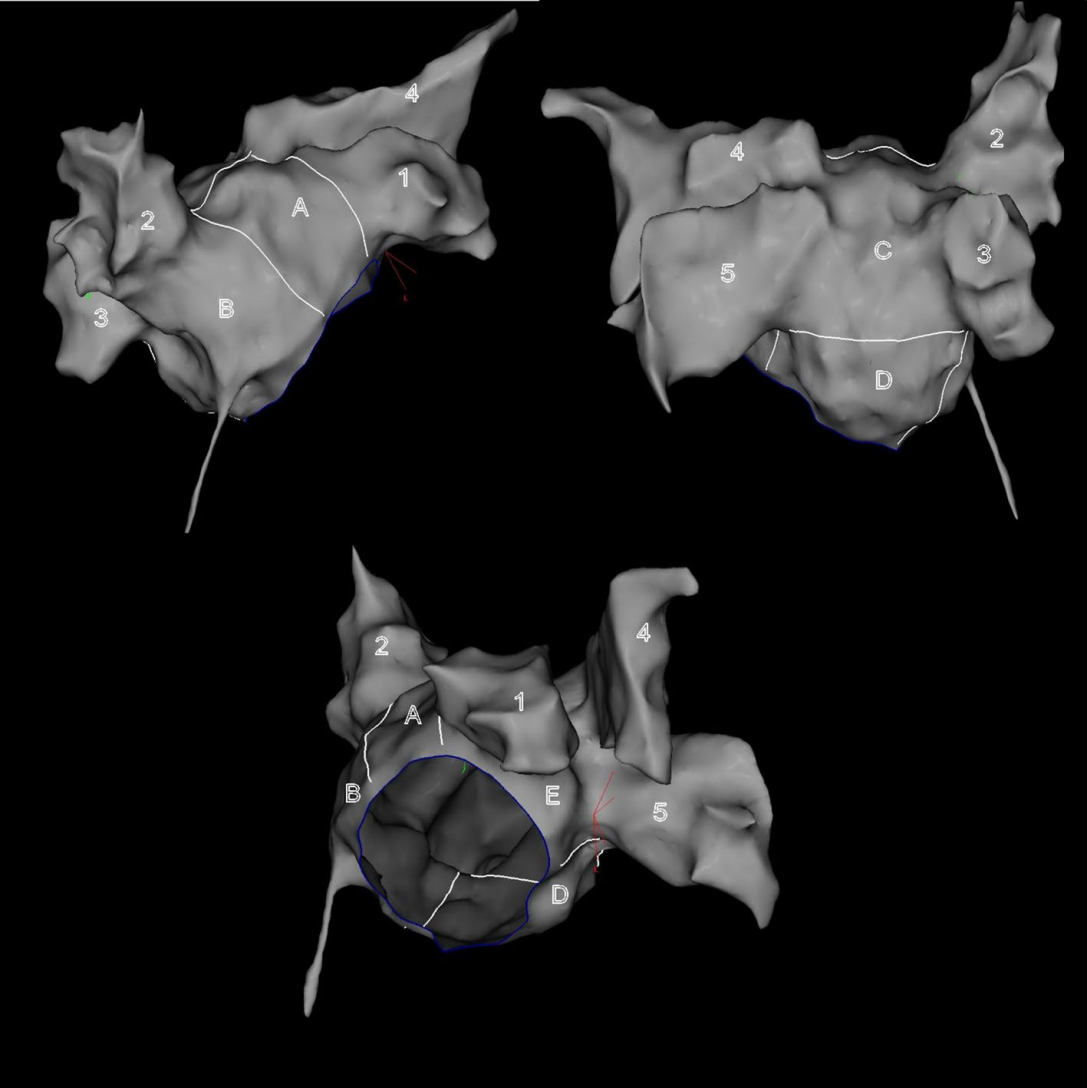
Predefined left atrial segmentation used for quantitative assessment of low-voltage areas. A = anterior wall, B = septum, C = posterior wall, D = inferior wall, E = lateral wall, 1 = left atrial appendage, 2 = right upper pulmonary vein, 3 = right lower pulmonary vein, 4 = left upper pulmonary vein, 5 = left lower pulmonary vein

An extension of all the areas showing low-voltage potentials at least 5 mm away from the ablation lesion set was measured with custom CARTO®3 system software. The global LVA burden was calculated as a sum of the all LVA and then expressed as a percentage of the TSA. The part of the PV inside ablation encirclement, LAA and an area adjacent to the fossa ovalis were excluded from the TSA calculations. The appendage was defined as an anatomical structure around the LAA orifice, determined internally from within the LA in the reconstructed shell. The extent of global LVA burden > 20% of the TSA was arbitrarily considered as severe on the basis of our observation that all detected LVA can be easily ablated if it occupies less than 20% of the TSA [2]. The body of LA was segmented into 5 areas: septum, anterior, posterior, inferior and lateral wall (Fig. 2). If LVA were identified within 3 out of 5 LA segments, it was considered a disseminated pattern of voltage-defined remodelling.

### Statistical analysis

Continuous data with non-normal distribution are expressed as median and IQR. The categorical variables are presented as values and percentages. Comparisons between groups were performed with the Mann–Whitney *U* test, the Chi^2^ test or the Wilcoxon signed-rank test, as appropriate. Univariate and multivariate logistic regression analyses were used to determine factors that were associated with the existence of LVA. Only variables with a *p* value of < 0.05 in univariate analysis were included for further evaluation in a multivariate model, using a stepwise forward regression. We used receiver-operating characteristic analysis to determine the optimal cutoff value for predicting the existence of LVA. The correlation between variables was assessed using a Spearman rank test. Statistical significance was accepted at *p* value < 0.05. The analysis was performed using Statistica software version 13.3 (StatSoft).

## Results

The baseline characteristics of the study population along with LAA and RAA electrical and mechanical function are summarised in Table 1. A significant difference between RAA and LAA AFCL (160 vs 145 ms, *p* < 000.1) was noted.

**Table 1:** Baseline characteristics

AF duration, months [range]	24 (12–36) [12–204]
Age, years [range]	63 (58–67) [37–79]
Females, *n* (%)	25 (18%)
BMI, kg/m^2^	30.1 (27.5–32)
Hypertension, *n* (%)	113 (81%)
Moderate mitral regurgitation, *n* (%)	40 (29%)
Moderate tricuspid regurgitation, *n* (%)	25 (18%)
Chronic coronary syndrome, *n* (%)	31 (22%)
Prior myocardial infarction, *n* (%)	10 (14%)
Heart failure, *n* (%)	35 (25%)
Ongoing tachycardiomyopathy, *n* (%)	10 (14%)
eGFR, ml/min/1.73m^2^	83 (69–93)
eGFR < 60 ml/min/1.73m^2^, *n* (%)	15 (11%)
Diabetes, *n* (%)	30 (22%)
Hyperthyroidism, *n* (%)	15 (11%)
CHA_2_DS_2_VASc ≤ 1, *n* (%)	48 (34%)
CHA_2_DS_2_VASc ≥ 2, *n* (%)	82 (59%)
CHA_2_DS_2_VASc ≥ 3, *n* (%)	48 (34%)
LVEF, %	60 (55–65)
LV internal diastolic diameter, mm	51 (48–56)
Enlarged LV [males LVd > 50.2 mm and females LVd > 45 mm], *n* (%)	105 (75%)
LA antero-posterior diameter, mm [range]	47 (45–50) [31–63]
LAAI, cm^2^/m^2^	13.1 (11.6–15.5)
LAVI, ml/m^2^	47.4 (37.7–59.5)
Enlarged LA [LAVI > 34 ml/m^2^], *n* (%)	118 (84%)
Severely enlarged LA [LAVI > 48 ml/m^2^], *n* (%)	61 (44%)
LAA peak inflow velocity, m/s	0.38 (0.28–0.56)
LAA peak outflow velocity, m/s	0.41 (0.31–0.6)
LAA low-peak inflow velocity, *n* (%)	14 (10%)
LAA low-peak outflow velocity, *n* (%)	6 (4%)
LAA depth, mm	29 (24–31)
RAA AFCL, ms	160 (145–170)
LAA AFCL, ms	145 (135–156)
Total AFCL, ms	150 (140–164)
AFCL gradient	0.9 (0.8–1.0)
RAA AFCL < LAA AFCL, *n* (%)	23 (16%)
RAA AFCL > LAA AFCL, *n* (%)	85 (61%)
RAA AFCL = LAA AFCL, *n* (%)	32 (23%)

*LV* left ventricle, *LVd* left ventricular internal diastolic diameter, *LA* left atrium, *LAVI* left atrial volume index, *LAAI* left atrial area index, *LAA* left atrial appendage, *RAA* right atrial appendage, *AFCL* AF cycle length

### The prevalence and distribution of LVA

Of the enrolled patients, all presented intact LAA. LVA [15 (7–30) cm^2^; 11 (5–22) % of the TSA] were detected in 54% (76/140) of the patients, whereas severe global LVA burden was present in 14% (20/140, 26% of the patients with LVA). Twenty-six percent of the patients (36/140) had documented LVA on the septum [8 (2.5–11) cm^2^], 31% (43/140) on the anterior wall [8 (3–12) cm^2^], 41% (58/140) on the posterior wall [7.5 (5–12) cm^2^], 25% (35/140) on the inferior wall [ 6 (4–10) cm^2^], and 14% (20/140) on the lateral wall [4 (3–7.75) cm^2^]. Twenty-nine percent of the patients (40/140, 53% of the patients with LVA) presented a disseminated pattern of remodelling. A single LA segment was affected in 16% (22/140) and limited to the posterior wall (82%), anterior wall (9%) and inferior wall (9%). The lateral LVA was only noted when there was already LVA elsewhere.

### A prediction of the existence of global and segmental LVA

Patients presenting LVA had significantly lower LAA peak inflow velocity and longer total AFCL. Patients with severe LVA had lower LAA peak inflow and outflow velocities, more often values presented as ≤ 0.2 m/s and had longer LAA and total AFCL. Patients demonstrating a disseminated LVA pattern had longer LAA and total AFCL (Table 2).

**Table 2:** Comparison between different LVA patterns

	LVA ( +) *n* = 76	LVA (–) *n* = 64	*p*	Severe LVA ( +) *n* = 20	Severe LVA (–) *n* = 120	*p*	Disseminated LVA pattern ( +) *n* = 40	Disseminated LVA pattern (–) *n* = 100	*p*
LAA peak inflow velocity	**0.34 (0.26–0.49)**	**0.4 (0.31–0.58)**	**.032**	**0.29 (0.17–0.39)**	**0.39 (0.3–0.57)**	**.005**	0.35 (0.23–0.57)	0.38 (0.3–0.55)	.29
LAA peak outflow velocity	0.4 (0.28–0.56)	0.46 (0.37–0.69)	.052	**0.28 (0.20–0.36)**	**0.47 (0.35–0.62)**	**.0002**	0.32(0.23–0.57)	0.45 (0.36–0.6)	.053
LAA low peak inflow velocity	8 (9%)	6 (9%)	.958	**4 (20%)**	**10 (8%)**	**.0257**	5 (13%)	9 (9%)	.41
LAA low peak outflow velocity	5 (7%)	1 (2%)	.190	**5 (25%)**	**1 (1%)**	**.0006**	4 (10%)	2 (2%)	.68
LAA depth	27 (24–31)	29 (25–31)	.5	28 (23–31)	29 (24–31)	.66	29 (23–31)	28.5 (24.30)	.94
RAA AFCL	160 (150–174)	155 (140–170)	.070	165 (150–175)	160 (143–170)	.176	160 (150–175)	160 (145–170)	.19
LAA AFCL	150 (138–163)	140 (133–150)	.086	**155 (145–180)**	**140 (135–150)**	**.006**	**150 (140–165)**	**140 (132–150)**	**.01**
Total AFCL	**155 (145–165)**	**150 (140–160)**	**.049**	**160 (150–185)**	**150 (140–161)**	**.02**	**160 (147–172)**	**150 (140–160)**	**.02**
AFCL gradient	0.93 (0.85–1)	0.93 (0.83–1)	.9	0.97 (0.87–1)	0.93 (0.83–1)	.157	0.97 (0.87–1)	0.92 (0.84–1)	.18
RAA AFCL > LAA AFCL	12 (16%)	11 (18%)	.794	5 (25%)	18 (15%)	.147	8 (20%)	15 (15%)	.9
RAA AFCL < LAA AFCL	49 (65%)	36 (56%)	.338	10 (50%)	75 (63%)	.256	19 (48%)	66 (66%)	.17
RAA AFCL = LAA AFCL	15 (19%)	17 (26%)	.372	6 (30%)	26 (22%)	.736	10 (25%)	22 (22%)	.7

Bold values indicate statistical significance at* p* < 0.05

*LVA* low-voltage areas. Other abbreviations as in Table 1

Using univariate logistic regression technique, it was found that neither LAA nor RAA functional features were associated with the prediction of the LVA.

Severe LVA burden was associated with slower RAA, LAA and total AFCL, decreased LAA filling and emptying velocities and the presence of low LAA emptying velocity. However, in the multivariate analysis, only LAA peak outflow velocity ≤ 0.2 m/s and LAA AFCL remained statistically significant (Table 3). A LAA AFCL cutoff value of 165 ms predicted severe LVA incidence with 50% sensitivity and 20% specificity.

**Table 3:** Predictors of LA remodelling

	Severe LA remodelling				Disseminated pattern of LA remodelling			
	Univariate		Multivariate		Univariate		Multivariate	
	OR (95% CI)	*p*	OR (95% CI)	*p*	OR (95% CI)	*p*	OR (95% CI)	p
LAA peak inflow velocity	**0.022 (0.001–0.823)**	**0.0199**			0.760 (0.099–5.837)	0.3		
LAA peak outflow velocity	**0.005 (0.000–0.211)**	**0.0048**			0.397 (0.062–2.555)	0.8		
LAA low peak inflow velocity	1.961 (1.008–3.817)	0.0627			1.518 (0.809–2.848)	0.2		
LAA low peak outflow velocity	**3.854 (1.578–9.418)**	**0.0025**	**5.180 (1.608–16.682)**	**0.006**	**3.279 (1.076–9.992)**	**0.016**		
LAA depth	1.018 (0.898–1.154)	0.785			1.014 (0.922–1.114)	0.8		
RAA AFCL	**1.019 (1.000–1.037)**	**0.0473**			1.013 (0.998–1.029)	0.08		
LAA AFCL	**1.027 (1.008–1.047)**	**0.0048**	**1.025 (1.004–1.045)**	**0.017**	**1.023 (1.006–1.041)**	**0.006**	**1.020 (1.003–1.038)**	**0.025**
Total AFCL	**1.030 (1.008–1.052)**	**0.0008**			**1.023 (1.004–1.043)**	**0.01**		
AFCL gradient	7.027 (0.173–284)	0.3			5.339 (0.269–106)	0.3		
RAA AFCL > LAA AFCL	1.431 (0.802–2.555)	0.242			1.278 (0.777–2.099)	0.3		
RAA AFCL < LAA AFCL	0.836 (0.511–1.368)	0.477			0.773 (0.522–1.144)	0.2		
RAA AFCL = LAA AFCL	0.979 (0.537–1.783)	0.943			1.245 (0.791–1.961)	0.3		

Bold values indicate statistical significance at* p* < 0.05

Abbreviations as in Table 1

Slower LAA and total AFCL and the presence of low LAA emptying velocity were found to be associated with the presence of a disseminated LVA pattern in the univariate model. In the multivariate model, only LAA AFCL left as the independent predictor (Table 3). Its value > 155 ms predicted a disseminated LVA pattern with 70% sensitivity and 45% specificity.

Further logistic regression analysis revealed that the same factors were found to be associated with the presence of LVA within the anterior and septal LA wall in the univariate model: decreased LAA peak outflow velocity [anterior OR 0.086 (95% CI 0.011–0.683), *p* = 0.02 septal OR 0.083 (95% CI 0.009–0.794), *p* = 0.03], LAA peak outflow velocity ≤ 0.2 m/s [anterior OR 3.144 (95% CI 1.032–9.575), *p* = 0.043 septal OR 2.196 (95% CI 0.720–5.507), *p* = 0.049], slower RAA [anterior OR 1.019 (95% CI 1.003–1.036), *p* = 0.02 septal OR 1.017 (95% CI 1.001–1.034), *p* = 0.035], slower LAA [anterior OR 1.037 (95% CI 1.016–1.058), *p *= 0.0004 septal OR 1.035 (95% CI 1.015–1.055), *p* = 0.0005], and slower total AFCL [anterior OR 1.036 (95% CI 1.014–1.059), *p* = 0.001 septal OR 1.033 (95% CI 1.012–1.0555), *p* = 0.0018]. In the multivariate model, only LAA AFCL was left as the independent predictor [anterior OR 1.035 (95% CI 1.014–1.045), *p* = 0.0008 septal OR 1.033 (95% CI 1.013–1.054), *p* = 0.001]. LAA AFCL value > 155 ms predicted the presence of LVA within anterior and septal LA wall with 78% sensitivity and 56% specificity. Only LAA peak outflow velocity ≤ 0.2 m/s was related to the presence of LVA within the lateral wall in the univariate assessment [OR 5.241 (95% CI 1.695–16), *p* = 0.0009]. No LAA or RAA functional features were associated with a prediction of LVA within posterior and inferior LA wall.

### LAA AFCL > 155 ms, LAA AFCL > 165 ms and LAA peak outflow velocity ≤ 0.2 m/s in LSPSAF population

LAA AFCL > 155 ms and > 165 ms had a positive correlation with the age of patients and RAA AFCL, whilst eGFR and CHA_2_DS_2_VASc ≤ 1 had an inverse correlation. RAA AFCL presented the highest level of association with LAA AFCL (Table 4). A RAA AFCL value > 165 ms predicted both the presence of LAA AFCL > 155 ms and > 165 ms with 70% sensitivity, 30% specificity and 80% sensitivity, 30% specificity, respectively. Moreover, patients presenting LAA AFCL > 155 ms but not > 165 ms less often showed CHA_2_DS_2_VASc score ≤ 1. No significant difference was found between any other factors, including AF duration, LA and LV size and function in patients with and without LAA AFCL > 155 ms and > 165 ms.

**Table 4:** Relationship between predictive factors of advanced LA voltage-defined remodelling and other variables

LAA AFCL > 155 ms		R Spearman	*p*	LAA > 155 ms ( +) *n* = 32	LAA > 155 ms (–) *n* = 108	*p*
	Age of patients	0.21	0.02	65 (60–68)	62 (57–66)	0.7
	eGFR	– 0.21	0.02	77 (64–85)	86 (71–95)	0.3
	CHA_2_DS_2_VASc ≤ 1	– 0.2	0.03	5 (17%)	43 (40%)	< 0.001
	RAA AFCL	0.47	< 0.001	170 (163–200)	158 (140–170)	0.009
LAA AFCL > 165 ms		R Spearman	*p*	LAA > 165 ( +) *n* = 24	LAA > 165 (–) *n* = 116	*p*
	Age of patients	0.21	0.02	65.5 (62–68.5)	62 (57–66)	0.36
	eGFR	– 0.23	0.01	75 (65–83)	86 (71–95)	1.0
	CHA_2_DS_2_VASc ≤ 1	– 0.23	0.01	2 (8%)	46 (40%)	0.09
	RAA AFCL	0.41	< 0.001	170 (160–200)	158 (140–170)	0.048
Low LAA outflow velocity		R Spearman	*p*	Low LAA outflow velocity ( +) [*n* = 6]	Low LAA outflow velocity (–) [*n* = 134]	*p*
	Age	0.19	0.04	67 (64–72)	63 (58–66)	0.9
	AF duration	0.18	0.046	60 (36–72)	24 (12–36)	< 0.005
	BMI	– 0.2	0.03	25.25 (22.9–28.6)	30.3 (27.8–32)	0.8
	eGFR	– 0.2	0.02	72 (57–75)	85 (71–94)	0.9
	LA antero-posterior diameter	0.18	0.04	47 (44–50)	51 (49–52)	0.5
	Chronic coronary syndrome	0.27	0.002	4 (67%)	27 (20%)	0.002
	Ongoing tachycardiomyopathy	0.25	0.006	2 (33%)	8 (6%)	0.3
	eGFR < 60 ml/min	0.18	0.047	2 (33%)	13 (10%)	0.01
	CHA_2_DS_2_VASc ≥ 3	0.24	0.007	5 (83%)	43 (32%)	< 0.001

Abbreviations as in Table 1

LAA peak outflow velocity ≤ 0.2 m/s had a positive correlation with the age of patients, AF duration, LA antero-posterior diameter, chronic coronary syndrome, ongoing tachycardiomyopathy, eGFR < 60 ml/min and CHA_2_DS_2_VASc ≥ 3 whilst BMI and eGFR values had an inverse correlation. The significant difference was limited to AF duration, BMI and eGFR values, as well as the presence of chronic coronary syndrome between patients with and without low LAA peak outflow velocity (Table 4). No correlation with LAA and RAA AFCL was found.

## Discussion

The present study evaluated whether the analysis of baseline LAA and RAA mechanical and electrical function could help to predict the presence of LVA in a large, unselected LSPAF population undergoing ablation. This is particularly important as LSPAF patients were regularly underrepresented in the vast majority of previous AF studies [2]. The following major observations were made:

1. LAA AFCL, RAA AFCL, LAA flow velocities and LAA depth did not predict the absolute presence of LA LVA.
2. LAA AFCL > 155 ms, LAA > 165 ms and LAA emptying velocity ≤ 0.2 m/s were predictive for the presence of advanced stages of voltage-defined LA fibrosis.
3. LAA AFCL > 155 ms was predictive for the existence of LVA within antero-septal LA segments whilst LAA emptying velocity ≤ 0.2 m/s within the lateral wall.
4. RAA AFCL > 165 ms was strongly related to the presence of LAA AFCL > 155 ms and > 165 ms.

### AFCL assessment

A relationship between arrhythmogenic sources operating in LSPAF and AFCL seems to be complex. Areas with shorter CL are thought to be the critical substrate for driving or maintaining the fibrillatory circuits in AF. However, accurate and reproducible AFCL measurements remain challenging due to complex AF activation patterns (low amplitude, multicomponent fractionated electrograms or depolarization of more than one overlapping muscle fascicle recorded simultaneously) [7]. The relatively unambiguous annotation of electrograms is likely to be achieved within atrial appendages, thereby facilitating AFCL assessment. The appendage is comprised of rigid pectinate muscles that are orientated in a whorl-like fashion throughout, with thin-walled myocardium interdigitating these raised regions [8]. The organised AF pattern within LAA is commonly observed as there are limited electrical breakthroughs, because the activity of the atrium must be filtered through the remaining fascicles to enter. This phenomenon could be also observed in the partially isolated PVs [9]. Whether mean RAA and LAA AFCL plays a role in predicting the extent of voltage-defined LA fibrotic substrate has not been previously investigated.

### LAA AFCL influence on the voltage-defined LA remodelling

It was clearly shown that mean baseline LAA AFCL helps characterise the extent of substrate ablation needed to achieve AF termination in patients with persistent AF during stepwise ablation. The shorter baseline mean AFCL, the more substrate ablation is needed due to higher number of areas with shorter CL that are thought to be the critical substrate for driving or maintaining the fibrillatory circuits. Mean AFCL > 157 ms was rarely observed in patients in whom AF could not be terminated. AFCL > 162.25 ms identified patients in whom AF is likely to be terminated by additional left-side ablation, whilst AFCL > 180.50 ms identified patients in whom AF is likely to be terminated only by PVI [7]. Whether LAA AFCL represents a global LA AFCL or rather segmental AFCL limited to the surrounding LA tissue (anterior and lateral wall) and correlates with the presence of critical local AF sources is unclear.

It was observed in this study that long LAA AFCL identifies patients with the advanced stage of voltage-defined LA fibrosis detected in sinus rhythm. AFCL > 155 ms identifies patient with LVA within at least 3/5 segments of LA, including the anterior and septal wall whilst > 165 ms patients with LVA surface area burden > 20% in the LA body. It seems that a progressive prolongation of LAA AFCL takes place with a fibrotic LA disease progression. Therefore, LAA AFCL reflects global, rather than segmental LA electrophysiological properties. However, LA antero-septal continuity, though not in the lateral wall, despite being in close proximity to LAA, seems to have a major influence on LAA AFCL, probably due to the complex fibre orientation at the junction between the LA and LAA orifice [10]. It is also thought that longer LAA AFCL translates to a lower number of areas critical for driving or maintaining AF due the larger extent of voltage-defined fibrotic areas. The lesser the extent of voltage-derived fibrosis, the shorter LAA AFCL and more ablation that is needed to terminate AF.

### RAA AFCL influence on the voltage-defined LA remodelling

There is a potential interplay between LAA and RAA, as both are directly connected through the Bachmann’s bundle. This runs in a sub-epicardial layer allowing conduction through the inter-atrial septum, approaches the LAA from the medial aspect of the atrial roof and branches both superiorly and inferiorly to encircle the neck of the LAA [11]. Therefore, we presumed that RAA AFCL, both directly and indirectly by total AFCL and gradient AFCL measurement may potentially unmask voltage-defined LA fibrotic substrate. Finally, it was found that RAA AFCL assessment does not translate into a prediction of LA LVA. However, long RAA AFCL > 165 ms indicates the possibility of the detection of voltage-derived remodelling of the LA, as it is associated with LAA AFCL > 155 ms and > 165 ms.

The variance between RAA and LAA AFCL, clear in our study, has been previously noted. It is usually thought that the atrium with shorter AFCL probably harbours driving or maintaining fibrillatory circuits, on the basis of observation that critical AF substrate is usually located at areas with shorter AFCL [7, 12]. However, Calo et al. showed that over 50% of patients in whom sinus rhythm was achieved by left-side ablation had shorter AFCL in RA than in LA and over 30% of patients in whom AF termination was achieved by right-side ablation had shorter AFCL in LA than in RA [13]. The exact reason which is responsible for this phenomenon is not clear. It is likely that there are multiple determinants of local intracardiac AFCL in association with atrial fibrotic remodelling.

It can be speculated that the presence of RA fibrotic areas, that were not assessed in this study, might have been at least a modifying factor to RAA AFCL. An increased level of fibrosis in the RAA, greater than in the LAA was observed in patients with structural heart disease undergoing cardiac surgery [14]. However it is well recognised that LA, not RA harbours most of the initiators or perpetuators of AF [7, 12, 13].

### Influence of LAA flow velocities on the voltage-defined LA remodelling

Clinical experience suggests that the evaluation of LAA function may serve as a clinically applicable surrogate of overall LA function, however, this is not well documented. Peak LAA outflow velocity represents LAA contraction and emptying, whilst peak LAA inflow velocity reflects LAA elastic recoil and relaxation [15].

Our study showed that only LAA emptying velocity was helpful in the identification of LA LVA. However, this was limited to the low LAA emptying velocity ≤ 0.2 m/s that was predictive for the presence of severe LVA burden and lateral LA wall involvement. The latter seems to be clearly explained because lateral LVA was only noted when there was already LVA elsewhere. Generally, our observations suggest that advanced fibrotic remodelling of LA body decreases LAA function. Furthermore, the low LAA emptying velocity was characterised by long AF duration, high CHA_2_DS_2_VASc score, low eGFR values and the presence of chronic coronary syndrome. However, many contributing factors beside LA LVA may have an influence on LAA flow velocities. Their values during AF are generally lower than those during sinus rhythm and highly variable. Increased LA pressure, commonly seen in the presence of LV systolic and/or diastolic dysfunction, pulmonary hypertension or valvular heart disease, is a major determinant of decreased LAA flow [15]. In our study cohort, 25% of patients presented heart failure and 29% were diagnosed with moderate mitral regurgitation. Therefore, it seems that the association of low LAA emptying velocity with other factors should be interpreted with caution.

In previous studies, it was found that LAA contraction velocity was an independent predictor of persistent AF termination through ablation [16]. Hori et al. observed that the presence of LVA on the anterior wall was associated with a low ≤ 0.2 m/s LAA emptying velocity in LSPSAF patients, with further reduction if LVA was located in the close proximity to the LAA orifice. The progression of atrial remodelling was considered to be a major contributing factor to this phenomenon, in addition to an ascending aorta being in direct contact with the anterior wall [17].

Our results differ from these data. A possible explanation includes the relatively low number of individuals with severely decreased LAA flow velocities (4%) observed in our study. It is worth noting that all 22 patients excluded from the ablation due to LAA thrombus detection presented low LAA emptying velocity. Moreover, in the study made by Hori et al., LVA were predominantly seen on the anterior wall, whereas the posterior wall was affected in the majority of cases in our study. A different LA voltage-mapping protocol and anatomical division of LA is likely responsible for the difference.

### The influence of LAA depth on voltage-defined LA remodelling

In AF, LAA develops negative remodelling resulting in dilatation and decreased contractility [15]. Differences that were observed in the anatomical parameters of LAA between persistent, paroxysmal and no AF, included LAA orifice diameters, depth and volume. An inverse correlation was observed between the LAA depth and LAA flow velocity. Positive correlations were observed between LAA depth, LA antero-posterior diameter and LAVI [18]. In our study, LAA depth did not predict the presence of LA LVA and did not correlate with low LAA flow velocities or long LAA AFCL.

### Why LAA functional features did not predict the absolute presence of LA LVA?

We can speculate that mild to moderate LA LVA might not have a great deal of impact on LAA functional features, even if located within close proximity to the LAA (antero-septal and lateral wall). The same effect may be potentially observed if LVA are limited to the posterior and/or inferior wall or when the vast majority of LVA are located within those regions, which are distant from LAA. Our observation that neither LAA nor RAA functional features predict LA LVA within the posterior and inferior wall supports this hypothesis.

### Regional distribution of LA LVA among the LSPAF population

Our report clearly differs from the other publications with regards to the distribution patterns of LA LVA among the persistent AF population. Comparison of our results with the previously published data is challenging due to the lack of standardised methodology for defining LVA, resulting in significant heterogeneity in voltage-mapping strategies among studies [2]. Moreover, persistent AF study cohorts are highly heterogeneous. The LSPAF population has been underrepresented in the vast majority of earlier research and patients unlikely to remain in sinus rhythm with very long AF duration, advanced age or severe left atrial enlargement were usually excluded. Our study cohort consists of a large unselected LSPAF population.

In the recent publications [19, 20], where voltage mapping was performed similarly to our protocol, the most frequent localization of LA LVA was the antero-septal region. In our population, LVA were most often located at the posterior wall, being also the most common single remodelling site. The lateral LA rarely displayed low-voltage values, analogous to our study, and has been never found to be a single remodelling site. Significant differences between the study populations and some in the mapping strategy might have been a major reason for achieving different results. On the other hand, our observations can be supported by the other recently published data [21]. The authors of the paper found that the fibrotic area detected by late gadolinium enhancement cardiac magnetic resonance is preferentially located at the posterior wall and floor around the antrum of the left inferior pulmonary vein. However, the exact underlying mechanism responsible for this phenomenon remains unclear.

## Conclusions

We have found that the LAA and RAA functional assessment may play a key role in the detection of advanced voltage-defined LA fibrosis among the LSPAF population, which is particularly important when voltage map-guided AF ablation procedures are applied. Although the AFCL within the appendages correlates with the extension of LA LVA, it requires invasive mapping of the atria. Therefore, a more important finding is that LAA flow velocity inversely correlates with the extent of LA LVA.

## Study limitations

1. The accuracy of LA voltage mapping might have been influenced by several factors, such as mapping during CS pacing, following PVI, using voltage cutoff values < 0.5 mV or due to functional voltage reduction related to the electrical stunning caused by long-lasting AF.
2. We cannot exclude that the overall LVA burden might have been altered due to the exclusion of patients not able to maintain sinus rhythm, presenting LAA thrombus or if another method of LVA detection had been applied.
3. Women were underrepresented in the study.
